# Effectiveness of Neuromuscular Training in Preventing Lower Limb Soccer Injuries: A Systematic Review and Meta-Analysis

**DOI:** 10.3390/jcm14051714

**Published:** 2025-03-04

**Authors:** Maria Stergiou, Alberto Lorenzo Calvo, Florian Forelli

**Affiliations:** 1 Department of Sports Medicine, Universidad Europea Madrid Real Madrid, 28055 Madrid, Spain; stergioumaria89@gmail.com (M.S.); alberto.lorenzo@upm.es (A.L.C.); 2 Haute-Ecole Arc Santé, HES-SO University of Applied Sciences and Arts Western Switzerland, 2800 Delémont, Switzerland; 3 Orthopaedic Surgery Department, Clinic of Domont, Ramsay Healthcare, @OrthoLab, 95330 Domont, France; 4 Société Française des Masseurs—Kinésithérapeutes du Sport Lab, 93380 Pierrefite sur Seine, France

**Keywords:** neuromuscular training, injury prevention, soccer players, FIFA 11+, lower limb injuries

## Abstract

**Background**: Soccer is associated with a high risk of injuries, mainly affecting the lower limbs, leading to significant consequences for player performance and career longevity. Neuromuscular training (NMT) has been proposed as an effective preventive strategy, but its impact varies across different populations and implementation strategies. **Methods**: A systematic review was conducted following PRISMA guidelines. A comprehensive search of PubMed, MEDLINE, and SPORTDiscus identified randomized controlled trials, cohort studies, and systematic reviews examining the effects of NMT on lower limb injury prevention in soccer players. Study quality was assessed using the Downs and Black tool, and injury incidence rate ratios were analyzed. **Results**: Eleven studies, encompassing over 10,000 soccer players, were included. NMT interventions, particularly FIFA 11+, significantly reduced injury rates compared to standard warm-ups. Higher adherence and coach education enhanced program effectiveness. Female players benefited more, particularly in ACL injury prevention. No significant differences were observed between shorter (10 min) and standard (20 min) NMT protocols. **Conclusions**: NMT effectively reduces lower limb injuries in soccer, with adherence and proper coaching being key determinants of success. Future research should optimize program design and long-term adherence strategies to maximize benefits across all player demographics.

## 1. Introduction

Soccer is the most widely played sport globally, with millions of participants at various competitive levels. While the sport offers substantial physical and psychological benefits, it also presents a considerable risk of injury, particularly in the lower extremities. Research indicates that lower limb injuries account for a significant proportion of total soccer-related injuries, with the knee (17%), ankle (25%), and hamstring (37%) being the most frequently affected areas [1,2]. These injuries can lead to prolonged recovery periods, decreased performance, and, in severe cases, early retirement from the sport [3].

Lower limb injuries are a major concern for soccer players, with hamstring strains, ankle sprains, and anterior cruciate ligament (ACL) injuries being among the most prevalent and debilitating. Hamstring strains are particularly common due to the high-intensity sprinting and sudden acceleration/deceleration involved in the sport, often leading to extended recovery periods and a high risk of recurrence [4,5]. Ankle sprains frequently result from rapid changes in direction, improper landings, or contact with an opponent, making them one of the leading causes of lost playing time [6,7]. Meanwhile, ACL injuries are among the most severe, typically occurring during pivoting movements, jump landings, or knee hyperextension, often requiring surgical intervention and prolonged rehabilitation [8,9,10].

However, there is still debate regarding the most effective approach, as factors such as gender, level of play, and program duration influence injury prevention outcomes [11,12,13]. Understanding the specific mechanisms of hamstring strains, ankle sprains, and ACL injuries and tailoring prevention programs accordingly could significantly improve injury outcomes and optimize player performance.

The growing concern regarding injury prevalence in soccer has prompted extensive research into preventive measures. Among the proposed strategies, neuromuscular training has gained significant attention due to its potential to enhance motor control, proprioception, and muscular strength, all of which contribute to injury mitigation [14,15]. Neuromuscular training encompasses various forms of intervention aimed at enhancing motor control, strength, balance, and movement efficiency to reduce injury risk. These programs can include balance training, plyometric exercises, strength conditioning, and proprioceptive drills. While some interventions focus on sport-specific movements, others emphasize general neuromuscular activation to improve overall stability and coordination. Neuromuscular training programs, such as the FIFA 11+, have been specifically designed to reduce lower limb injury incidence by integrating exercises that focus on dynamic stability, strength, and coordination [16,17,18,19]. Studies have demonstrated that teams implementing the FIFA 11+ experience a substantial decline in injury rates compared to those following conventional warm-up routines [20,21,22]. However, despite these promising findings, inconsistencies in adherence and variations in program execution have raised concerns about its practical effectiveness across different age groups, sexes, and competitive levels [23,24].

While previous studies have highlighted the effectiveness of neuromuscular training, several gaps remain in the literature. First, there are limited data regarding the influence of intervention frequency and program duration on injury prevention outcomes [25]. Second, the role of coach education in optimizing program implementation remains underexplored [23]. Furthermore, most existing studies have primarily focused on male athletes, leaving a significant knowledge gap regarding the effectiveness of neuromuscular training in female soccer players, who tend to experience higher rates of anterior cruciate ligament injuries [26,27].

Given these gaps, this systematic review aims to critically evaluate the impact of neuromuscular training programs on the prevention of lower limb injuries in soccer players. Specifically, this study seeks to examine the effectiveness of various neuromuscular training interventions in reducing lower limb injury rates. It will also assess the influence of program adherence, duration, and frequency on injury prevention outcomes while investigating the impact of coach education on program efficacy. Finally, it will compare the effectiveness of neuromuscular training between male and female soccer players. By addressing these aspects, this review aims to provide a comprehensive synthesis of the existing evidence and offer practical recommendations for optimizing neuromuscular training interventions in soccer injury prevention programs.

## 2. Methods

### 2.1. Study Design

This systematic review was conducted following the Preferred Reporting Items for Systematic Reviews and Meta-Analyses (PRISMA) guidelines for systematic reviews and meta-analyses [28]. The research focused on investigating the effectiveness of neuromuscular training programs in preventing lower limb injuries among soccer players. The methodology was designed to ensure a rigorous and systematic approach to analyzing existing studies, minimizing bias, and enhancing reproducibility [29].

### 2.2. Search Strategy

A comprehensive literature search was performed using electronic databases such as PubMed, MEDLINE, and SPORTDiscus. The search strategy included relevant keywords such as “neuromuscular training”, “injury prevention”, “soccer players”, “FIFA 11+”, “lower limb injuries”, and “ACL injury prevention”. Boolean operators (AND, OR) were used to refine the search results. Only peer-reviewed articles published in English between 2000 and 2023 were considered. Additional studies were identified through cross-referencing relevant articles found in the initial search [30].

To ensure a comprehensive review, we conducted an iterative search process. This involved refining search terms and reviewing citation lists from key articles to identify relevant studies that might have been overlooked in the initial search (Table 1).

This model helped streamline the identification of relevant studies, ensuring that only those directly related to neuromuscular training and injury prevention in soccer were included.

### 2.3. Inclusion and Exclusion Criteria

The inclusion criteria for this review were: studies investigating the impact of neuromuscular training on lower limb injury prevention in soccer players, randomized controlled trials, cohort studies, and systematic reviews [31]. Studies had to report injury incidence rates as an outcome and include male and/or female soccer players at various competitive levels. Only studies with clearly defined intervention protocols and measurable outcomes were included.

Exclusion criteria included studies focusing on non-soccer athletes, case reports, letters, studies without full-text availability, and studies that did not provide quantitative data on injury rates. Additionally, studies that lacked appropriate control groups or used subjective assessment methods without objective injury reporting were excluded to ensure methodological rigor.

### 2.4. Study Selection and Data Extraction

Following the initial search, duplicate articles were removed. Titles and abstracts were screened for relevance, and full-text articles were subsequently reviewed for final inclusion. Two independent reviewers assessed each study to reduce selection bias. Any disagreements were resolved through discussion or consultation with a third reviewer.

Data extracted from each study included sample size, participant demographics, type and duration of intervention, frequency of application, reported injury outcomes, and statistical measures such as risk ratios or confidence intervals. Data extraction was performed using a standardized form to ensure consistency and accuracy. Data extracted from each study included sample size, participant demographics, type and duration of intervention, frequency of application, reported injury outcomes, and statistical measures such as risk ratios or confidence intervals. Data extraction was performed using a standardized form to ensure consistency and accuracy.

### 2.5. Quality Assessment

To assess the quality and risk of bias in the selected studies, the Downs and Black quality assessment tool was used [31]. This tool evaluates study quality across multiple domains, including reporting, external validity, internal validity (bias and confounding), and statistical power. Studies were categorized as having poor, moderate, or high methodological quality based on predefined criteria [32].

To ensure objectivity, two independent reviewers conducted the quality assessment. Studies with low scores (≤14) were classified as poor quality, those with scores between 15 and 19 were considered moderate quality, and those scoring above 20 were deemed high quality. Discrepancies between reviewer assessments were resolved through consensus.

### 2.6. Statistical Analysis

A meta-analysis was conducted where feasible, using injury incidence rate ratios (IRRs) as effect measures. Statistical heterogeneity was assessed using the I^2^ statistic, which quantifies variability across studies that is not attributable to chance [33]. An I^2^ value greater than 50% was considered indicative of substantial heterogeneity, warranting a random-effects model [34]. For studies with low heterogeneity, a fixed-effects model was applied.

When meta-analysis was not possible due to high variability in study methodologies, intervention designs, or outcome measures, a narrative synthesis was conducted. This approach involved summarizing findings from individual studies, identifying patterns, and discussing methodological differences that might explain variations in results.

This methodology ensures a rigorous and systematic approach to evaluating the role of neuromuscular training in soccer injury prevention, providing evidence-based insights for practitioners and researchers in sports science and medicine. The combination of meta-analysis and narrative synthesis allows for a holistic understanding of the evidence, contributing to informed decision-making in injury prevention strategies.

## 3. Results

### 3.1. Study Selection

**Figure 1:**
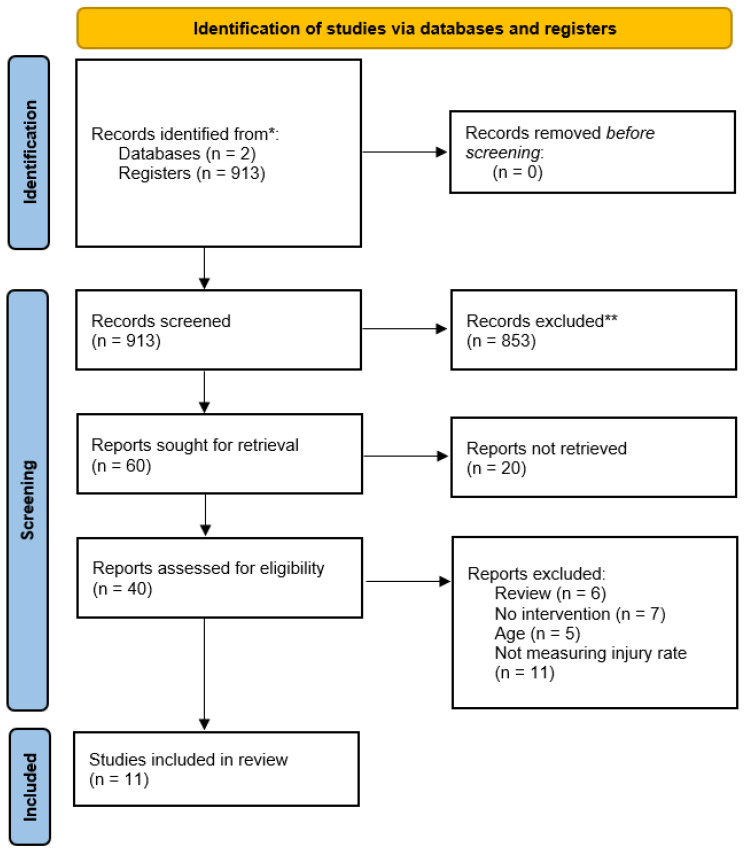
The PRISMA flow chart followed for studies selection. (*, ** according exclusion criteria).

The initial search yielded 913 studies. No duplicates were found and no filters were applied, so none of the records were removed before the screening process. On the first step of selection process, after the screening of titles and abstracts, 873 articles were removed for not complying with the selection criteria, and 60 studies remained for full text screening. Of those, the full text was not available for 20 articles, leaving 40 full texts for the next step of the screening process. Based on the full text screening, six articles were excluded in a review, seven studies did not contain neuromuscular training as intervention, five were excluded because the age of population was not in the range determined as eligible, and a further eleven were excluded because they did not study occurrence of injuries as an outcome. Based on that, only 11 studies were found to be eligible for inclusion in the present systematic review. The selection process is described in the PRISMA flow chart below (Figure 1).

### 3.2. Study Charactristics

This systematic review included 11 studies, with a total sample of more than 10,000 soccer athletes. The basic study characteristics are described in Table 2.

Most studies included only men [11,13,14,24,25], three studies included only women [21,23,35,36], while two studies included soccer players of both sexes [26,37]. Interestingly, six of the eleven studies included athletes with a mean age <18 [21,23,25,36,37,38] and another three studies included athletes with average age 18–25 years [22,24,35]. The studies by Finch et al. and Åman et al. do not give detailed information about the age of the studied population, except that it concerned athletes over 18 years of age [26,39]. The sample size also varied significantly, ranging between 37 and 4564 participants (Åman et al. did not provide a specific number of athletes studied) [26].

The 11 studies selected for inclusion in this systematic review were published between 2008 and 2020. Regarding the country where the studies were carried out, it is observed that most of the studies were carried out in American countries, with three studies having been carried out in the United States of America [22,24,35] and three in Canada [23,37]. Moreover, four studies were identified that have been carried out in Europe [21,25,26,36], one in Africa—specifically in Nigeria [38], and one in Australia [39].

**Table 2:** Basic study characteristics.

Authors (Year)	Study Design	Study Duration	Country	Participants	Intervention	Measurements	Incidence Rate
Soligard et al. (2008) [21]	Randomized controlled trial	8 months (one league season)	Norway	1892 female youth soccer players (Exp: n = 1055, 13–17 years, 15.4 ± 0.7; Control: n = 837, 13–17 years, 15.4 ± 0.7)	FIFA 11+ program (initial running drills, exercises, final running drills); 20 min/session, 2–5 times/week	Injury occurrence (RR, CI)	Exp: RR = 0.71, CI 0.49–1.03; lower overall injury risk (0.68, 0.48–0.98), overuse injuries (0.47, 0.26–0.85), severe injuries (0.55, 0.36–0.83)
Emery and Meeuwisse (2010) [37]	Randomized controlled trial	1 year	Canada	744 soccer players (Exp: n = 380, 57.6% male, 42.4% female, ages 13–18; Control: n = 364, 31% male, 69% female, ages 13–18)	Standardized warm-up + neuromuscular and home-based balance training; 15 + 5 + 15 min/session, ≥3 times/week	Injury rates per 1000 player-hours	Exp: 2.08; Control: 3.35; All injuries IRR: 0.62 (CI = 0.39–0.99)
Waldén et al. (2012) [36]	Randomized controlled trial	7 months (one season)	Sweden	4564 female adolescent soccer players (Exp: n = 2479, 12–17 years; Control: n = 2085, 12–17 years)	Neuromuscular warm-up focusing on knee control and core stability; 15 min/session, 2 times/week	ACL injury rate	Exp: 64% reduction (RR 0.36, CI 0.15–0.85)
Grooms et al. (2013) [35]	Cohort study	2 years	USA	41 male collegiate soccer players (Exp: n = 30, 18–25 years, 20.3 ± 1.6; Control: n = 34, 18–25 years, 20.0 ± 2.4)	FIFA 11+ program; 3 progressive difficulty levels, 20 min/session, 5–6 times/week	Injury rates per 1000 exposures, days lost	Exp: 2.2, 52 days lost; Control: 8.1, 291 days lost; 72% injury rate decrease (RR = 0.28, CI = 0.09–0.85, *p* < 0.01)
Steffen et al. (2013) [23]	Randomized controlled trial	1 soccer season	Canada	226 female youth soccer players (Comprehensive: n = 78, 13–18 years; Regular: n = 68, 13–18 years; Control: n = 80, 13–18 years)	FIFA 11+ program with varying implementation support	Injury risk	Comprehensive vs. Control: IRR = 0.28 (CI = 0.10–0.79)
Owoeye et al. (2014) [38]	Randomized controlled trial	6 months (one league season)	Nigeria	416 male youth soccer players (Exp: n = 212, 17.80 ± 0.94; Control: n = 204, 17.49 ± 1.10)	FIFA 11+ program; 3 progressive difficulty levels, 20 min/session, ≥2 times/week	Injury reduction rate	Exp: 41% decrease (RR = 0.59, CI = 0.40–0.86, *p* = 0.006), 48% decrease in lower extremity injuries (RR = 0.52, CI = 0.34–0.82, *p* = 0.004)
Silvers-Granelli et al. (2015) [22]	Randomized controlled trial	1 competitive collegiate soccer season	USA	1525 collegiate male soccer players (Exp: n = 675, 20.40 ± 1.66; Control: n = 850, 20.68 ± 1.46)	FIFA 11+ program; 3 progressive difficulty levels, 20 min/session, 3 times/week	Injury rates and days lost	Exp: 285 injuries (IR = 8.09), 10.08 ± 14.68 days lost; Control: 665 injuries (IR = 15.04), 13.2 ± 26.6 days lost (*p* = 0.007)
Finch et al. (2015) [39]	Randomized controlled trial	2 years	Australia	1564 male soccer players (Exp: n = 679, >18 years; Control: n = 885, >18 years)	Neuromuscular and biomechanical exercises; 2 times/week	Lower limb injury rates	Exp: IRR = 0.78 (CI 0.56–1.08, *p* = 0.14); knee injuries IRR = 0.50 (CI 0.24–1.05, *p* = 0.07)
Silvers-Granelli et al. (2018) [24]	Secondary analysis of RCT	1 competitive collegiate soccer season	USA	675 collegiate male soccer players, 20.40 ± 1.66 years	FIFA 11+ program; 3 progressive difficulty levels, 20 min/session, 3 times/week	Compliance vs. injury rate	Low compliance: IR = 10.34; Moderate: IR = 8.6; High: IR = 6.4 (*p* = 0.004)
Åman et al. (2018) [26]	Descriptive epidemiological study	9 years	Sweden	-	Swedish Knee Control Programme	Cruciate ligament and knee injury rates	Males: 6% decrease (RR = 0.94, CI 0.89–0.98); Females: 13% decrease (RR = 0.87, CI 0.81–0.92)
Rahlf and Zech (2020) [25]	Randomized controlled trial	1 soccer season	Germany	342 male youth soccer players (Exp1: n = 175, 14.5 ± 1.5; Exp2: n = 167, 15.3 ± 1.4)	FIFA 11+ program; 3 progressive difficulty levels, 2 times/week	Injury rates per 1000 h	INT10: 6.37; INT20: 7.20; IRR = 1.03 (CI 0.59–1.79)

Note: NMT—Neuromuscular Training, FIFA 11+—FIFA 11+ Injury Prevention Program, RR—Relative Risk, CI—Confidence Interval, IRR—Incidence Rate Ratio, ACL—Anterior Cruciate Ligament, MC—Moderate Compliance, HC—High Compliance, LC—Low Compliance, INT20—Intervention 20 min (Long version of FIFA 11+ program), INT10—Intervention 10 min (Short version of FIFA 11+ program.

Diversity is also observed in the type of studies that the researchers chose to carry out to find out if neuromuscular training can help avoid injuries to the lower limbs. Most chose to conduct a randomized controlled study [21,22,23,24,25,36,37,38,39], which has been described by many as the gold standard of clinical trials [40] due to the reduced bias and increased reliability of its results. Also, a cohort study [35] and a descriptive epidemiological study [26] were identified. The duration of the studies included in the review varied significantly, ranging from 6 months to 9 years, with the majority of studies lasting one competitive season [21,22,23,24,25,36,38].

The most common neuromuscular training was the FIFA 11+ program [21,22,23,24,25,35,38], a warm-up program consisted of three parts: initial running drills, exercises, and final running drills. The duration of the program is 20 min and there are three levels of progressive difficulty, which is elevated as the athletes become more familiar with it. As we can see in Table 2, in five of seven studies [21,22,24,35,38] that investigated the efficacy of the FIFA 11+ program in injury prevention, they compared it with a standardized warm-up program. Steffen et al. compared the efficacy of three different levels of coach education in the FIFA 11+ program [23]. The coaches in the control group were given online access to the website for the 11+ program. For the 11+ program’s preseason coach training, the regular group coaches were given 11+ materials (video, a poster detailing the exercises, and website information). The complete group coaches offered everything mentioned above in addition to a designated 11+ study physiotherapist who instructed the players in the 11+ program and took part once a week in practices to support the proper technique and progression of the program’s components. On the other hand, Rahlf and Zech compared the efficacy of the FIFA 11+ program’s suggested duration (20 min) versus a shorter duration (10 min) [25]. The shorter timing was achieved by shortening the duration or quantity of repetitions for each exercise. The frequency of conducting the FIFA 11+ program was at least two times to a maximum of six times per week.

Other types of neuromuscular training were also evaluated. Emery and Meeuwisse compared the efficacy of a standardized warm-up program with a 15 min duration versus the addition of 5 min neuromuscular training and a 15 min home-based balance training program [37]. Both the control and the intervention were conducted in the program at least three times per week. Waldén et al. compared a 15 min neuromuscular warm-up program with exercises emphasizing knee control and core stability with a frequency of twice per week to a regular warm-up [36]. Finch et al. selected two different programs to compare: the intervention group tried a training program of evidence-based neuromuscular and biomechanical exercises specifically targeted at minimizing lower limb injuries, whereas the control group performed a “mock” program of exercises similar to those usually performed [39]. Both programs were performed twice a week. Finally, Åman et al. conducted a descriptive epidemiological study comparing the frequency of injuries before and after implementing the Swedish Knee Control Program, a neuromuscular training program focusing on the prevention of injuries [26], nationwide.

### 3.3. Results of Individual Studies

#### 3.3.1. FIFA 11+ Program Versus Standardized Warm-Up

The oldest study found was the one of Soligard et al., who conducted a randomized controlled trial in Norway in order to compare the injury rate of female youth soccer players, implementing the FIFA 11+ program (n = 1055) with a standardized warm-up (n = 837) [21]. A total of 121 players of the 1055 in the intervention group and 143 players of the 837 in the control group mentioned injuries, resulting in relative risk (RR) = 0.71 (95% CI 0.49–1.03). This study also showed that players implementing the FIFA 11+ program have a lower risk of injuries overall (0.68, 0.48–0.98), overuse injuries (0.47, 0.26–0.85), and severe injuries (0.55, 0.36–0.83) [21].

Grooms et al. carried out a cohort study in the USA of 41 male collegiate soccer players where they compared the injury rate at the first season before the introduction of the FIFA 11+ program while athletes were using a standardized warm-up (n = 34), versus the rate at the second season, where they used the FIFA 11+ program (n = 30). The injury rate in the first season was 8.1 injuries per 1000 exposures and 291 days lost, while in the second season, the rate diminished to 2.2 per 1000 exposures and the days lost decreased to 52. Calculating the RR of lower extremity injury, authors point out that the intervention decreased the injury rate by 72% (RR = 0.28, 95% CI = 0.09–0.85), also having a great impact on the amount of time lost due to lower extremity injury (*p* < 0.01) [35].

Owoeye et al. also carried out a randomized controlled trial to compare the FIFA 11+ program (n = 212) with a usual non-structured warm-up (n = 204) in 416 Nigerian male youth soccer players. The intervention group showed a 41% decrease (RR = 0.59, 95% CI = 0.40–0.86, *p* = 0.006) in the overall rate of injury and a 48% decrease (RR = 0.52, 95% CI = 0.34–0.82, *p* = 0.004) in all lower extremity injuries compared to the control group [38].

Silvers-Granelli et al. conducted a randomized controlled trial with 1525 collegiate male soccer players in USA to compare the efficacy of FIFA 11+ program (n = 675) versus a standardized warm-up (n = 850). The control group reported 665 injuries (incidence rate (IR) = 15.04 injuries per 1000 exposures) and 13.2 ± 26.6 days lost of the season, while the intervention group reported 285 injuries (IR = 8.09 injuries per 1000 exposures) and 10.08 ± 14.68 days lost of the season (*p* = 0.007) [22].

#### 3.3.2. Difference in FIFA 11+ Program Dose

Steffen et al. made a comparison of the efficacy of three dosages of soccer coaches’ education in the FIFA 11+ program, only allowing online access to the website for the 11+ program (control group), introducing a preseason coach workshop with 11+ content for the 11+ program (regular group), or all of the above, in addition to a designated 11+ study physiotherapist who instructed the players in the 11+ program and was expected to take part in a practice session once a week to facilitate proper technique and progression of the program components (comprehensive group). Results showed that injury risk decreased in players trained by coaches randomly assigned to comprehensive group (IRR = 0.28, 95% CI = 0.10–0.79) compared to those in other groups [23].

The Silvers-Granelli’s research team published another article in 2018 examining how variations in compliance may have an influence on injury rates and time lost from injury. To do that, they separated the intervention group into three subgroups based on compliance to the intervention. Low compliance (LC) was defined as 1–19 doses/season, moderate compliance (MC) as 20–39 doses/season and high compliance (HC) as >40 doses/season. When researchers compared the injury rate between the three groups, they found a significant difference (*p* = 0.04). Specifically, the LC group had a mean injury rate of 13.25 (95% CI = 9.82–16.68), while the HC group had 8.33 (95% CI = 6.05–10.62) (*p* = 0.02). The mean injury rate of the MC group (11.21, 95%CI: 9.38–13.05) was not significantly lower than the LC group (*p* = 0.29), but was significantly higher than the HC group (*p* = 0.05). Statistical analysis showed that compliance was markedly inversely correlated to the injury rate (*p* = 0.004) and the number of days missed (*p* = 0.012) [24].

Rahlf and Zech carried out a randomized controlled trial with 342 male youth soccer players in Germany to compare the effectiveness in reducing injury rate when implementing the classic FIFA 11+ program (duration 20 min) (INT20) (n = 167) versus a shorten 10 min version (INT10) (n = 175). Comparing the injury rate between two groups, there was no discernible group difference (INT10: 6.37 per 1000 h, INT20: 7.20 per 1000 h, IRR = 1.03, 95% CI = 0.59–1.79) [25].

#### 3.3.3. Other Neuromuscular Training Programs

Emery and Meeuwisse carried out a randomized controlled trial with 744 soccer players in Canada to compare the effectiveness of a standardized 15 min warm-up program (n = 380) with the addition of a 5 min neuromuscular training and a 15 min home-based balance training program (n = 364). Comparing the two groups, the intervention group had 2.08 injuries/1000 player-hours, while the control group had 3.35 injuries/1000 player-hours. The IRR calculated as 0.62 (95% CI = 0.39–0.99) for all injuries, 0.57 (95% CI = 0.35–0.91) for acute onset injury, 0.68 (95% CI = 0.42–1.11) for lower extremity injuries, 0.5 (95% CI = 0.24–1.04) for ankle sprain injuries and 0.38 (95% CI = 0.08–1.75) for knee sprain injuries [37].

Waldén et al. compared a neuromuscular warm-up program with exercises focusing on knee control and core stability (n = 2479) with a standardized warm-up program (n = 2085) through a randomized controlled trial in Sweden. The results of this study showed that the intervention diminished the frequency of anterior cruciate ligament injuries by 64% (RR 0.36, 95% CI = 0.15–0.85), with the absolute rate difference being −0.07 (95% CI −0.13–0.001) per 1000 player-hours, in the intervention group’s favor. On the other hand, there was no discernible decline in the frequency of serious knee injuries (>4 weeks’ absence) or acute knee injury [36].

Finch et al. conducted a randomized controlled trial with 1564 Australian male soccer players to contrast a ‘sham’ program of exercises similar to those typically practiced during training (n = 885) with a program of evidence-based neuromuscular and biomechanical exercises specifically targeted at minimizing lower limb injuries (n = 679). Players randomized at the intervention group had fewer lower limb (IRR = 0.78, 95% CI 0.56, 1.08, *p* = 0.14) and knee injuries (IRR = 0.50, 95% CI = 0.24 = 1.05, *p* = 0.07) than the control group [39].

Åman et al. conducted a descriptive epidemiological study in Sweden, comparing the injury rate before and after the introduction of Swedish Knee Control Program. The research showed that the incidence of cruciate ligament decreased for both male (from 2.9 to 2.4 per 1000 player-years) and female players (from 4.9 to 3.9 per 1000 player-years) after introducing the Swedish Knee Control Program. Also, they showed a decrease in overall incidence of knee injuries in both sexes (males: from 5.6 to 4.6 per 1000 player-years, females: from 8.7 to 6.4 per 1000 player-years). These results mean that the nationwide implementation of the program resulted in a decrease in the incidence of cruciate ligament injuries by 6% (RR = 0.94, 95% CI = 0.89–0.98) in male players and 13% (RR = 0.87, 95% CI = 0.81–0.92) in female players and a decrease in the incidence of knee injuries by 8% (RR = 0.92, 95%, CI = 0.89–0.96) and 21% (RR = 0.79, 95% CI = 0.75–0.83), respectively (*p* < 0.01 for all) [26].

### 3.4. Quality Assessment and Risk of Bias

Table 3 shows the quality assessment of selected studies based on the Downs and Black (DB) quality assessment tool scale [31]. According to the categorization proposed by Hooper et al., two studies have poor quality [26,35], one study has average quality [39], while the remaining studies have good quality [21,22,23,24,25,36,37,38]. None of the studies in this review had a high score which refers to excellent quality.

Based on Table 3, it is observed that some criteria are not met by any study (criteria 8 and 14). Criterion 8 refers to whether the researchers reported the adverse events of the intervention. The studies selected in this review mainly concern the modification of the warm-up, where the most obvious adverse event that could occur is the frequency of injuries, which is, however, also the main variable under consideration. Another parameter that could be considered is perhaps the ease of carrying out the intervention or the level of fatigue induced by the intervention program versus the control program in the athletes; however, neither was assessed in the studies identified. Criterion 14 concerns the blinding of participants, which, due to the nature of the intervention, was not possible to achieve and was thus not expected to be met by any study.

Since the majority of studies show good methodological quality (Downs and Black score ≥20), the results of the studies can be considered reliable.

## 4. Discussion

One of the most popular sports in the world is soccer and lower limb injuries are the most common type of injury encountered in it [2]. The purpose of this work was to investigate the effectiveness of neuromuscular training in the prevention of lower limb injuries in soccer players, through a systematic review. The international literature search yielded 11 studies that were included in the review as they met the criteria set. These studies investigated either the effectiveness of neuromuscular training as a warm-up versus a conventional warm-up program [21,22,26,35,36,37,38,39], or the effectiveness of neuromuscular training at different dosages that differed in the frequency of use of the program [22] or its duration [25], while a study was found that investigated the effectiveness of the program depending on the level of education of the coaches [23].

FIFA 11+ is the most common neuromuscular training program in use today. The program consists of three stages and fifteen exercises that are performed in a particular order [41]. Comparing the effectiveness of the application of this program against the conventional warm-up program, it was observed that in all cases the rate of lower limb injuries significantly decreased [21,22,35,38], showing that this is an effective intervention. Moreover, similar results were observed for the other types of neuromuscular training investigated in the studies of Emery and Meeuwisse, Waldén et al., Finch et al. and Åman et al., all of which showed a reduction in injuries following a neuromuscular program implementation [26,36,37,39].

A comment that should, however, be highlighted is that of the studies comparing FIFA 11+ program with a conventional warm-up program, only one included women [21], while the rest exclusively included males [22,35,38]. Although Soligard et al. found a decrease in the frequency of injuries among women as well, there is a clear lack of data on the effectiveness of the program in women [21]. Regarding the other neuromuscular training programs studied, Emery and Meeuwisse and Åman et al. included both men and women, while Waldén et al. and Finch et al. only included men [26,36,37,39]. Interestingly, Åman et al., who compared the effectiveness of the program between the two sexes, observed that in women, the program resulted in a more notable decrease in the number of injuries, with cruciate ligament injuries being reduced by 6% in men and 13% in women, while knee injuries were reduced by 8% in men and 21% in women [26]. The fact that injuries occur more frequently in female athletes than in male competitors may help to explain this, leading the injury prevention program to be more important for this population and presenting a greater efficiency [42,43,44].

Although neuromuscular training appears to be successful in protecting soccer athletes from injury, the question remains whether the dosage of the program can affect its effectiveness. Silvers-Granelli et al. compared the incidence of injuries between groups, showing the different adherence rates to the program [24]. They observed that adherence to the program showed a statistically significant negative correlation to the frequency of injuries and also to the days lost due to them. This shows that the more this program is used, the more its effectiveness increases.

Another interesting study is that of Rahlf and Zech who compared the effectiveness of the application of the classic FIFA 11+ program, lasting 20 min, against a shortened form, lasting 10 min [25]. The study showed that the two programs did not show statistically significant differences in terms of their effectiveness in preventing injuries, showing that even a short warm-up based on neuromuscular training can be effective. This is a very important finding, since the duration of the program may in many cases act as a deterrent to the implementation of the program, as the coaches consider that they must implement it either in its entirety or not at all. This assumption is overturned by Rahlf and Zech and they believe that is information that should be widely known [25].

Although FIFA 11+ is now a well-known neuromuscular training program, the familiarity among coaches with it is not the same. Steffen et al. investigated the efficiency of various coach educational levels in the program in terms of injury prevention [45]. All coaches had online access to the 11+ program website, but some coaches also received one preseason coach workshop and additional informational material about the program, while other teams also received a designated 11+ study physiotherapist who instructed the players in the 11+ program and was expected to take part once a week in a practice session to facilitate proper technique and progression of the program’s elements. It was observed that the teams in the latter category presented a significantly lower probability of injury compared to the first two, which shows that the correct training of the coaches and familiarity with the program plays a decisive role in its correct implementation and, by extension, in its effectiveness. This should be taken into account and more intensive training sessions should be carried out for soccer coaches, while supervision, at least for a period of time from the start of the implementation of the program by an expert is vital to correct any mistakes or omissions.

While this systematic review highlights the short-term benefits of NMT in reducing lower limb injuries among soccer players, an important aspect that remains unexplored is its long-term effectiveness. Most studies included in this review assess injury prevention outcomes only during the intervention period, without evaluating whether these benefits persist once the program is no longer actively implemented. This represents a significant gap in the literature, as understanding the long-term impact of NMT is crucial for optimizing injury prevention strategies.

Future studies should incorporate follow-up assessments beyond the immediate intervention phase to determine whether the protective effects of NMT are sustained over time. This would help identify whether players continue to apply learned neuromuscular strategies in their training and competition or if the benefits diminish after discontinuation. Additionally, research should explore whether periodic booster sessions or continued partial adherence to NMT programs could help maintain injury reduction benefits in the long term.

Another key consideration is the role of habit formation and motor learning in sustaining the advantages of NMT. It is possible that repeated exposure to NMT exercises leads to neuromuscular adaptations that persist beyond the intervention, reducing injury risk even after structured training ceases. Conversely, without reinforcement, these adaptations might fade over time, highlighting the need for long-term monitoring and maintenance strategies.

Furthermore, studies should investigate whether players who have undergone NMT programs experience lower reinjury rates in subsequent seasons compared to those who have never participated in such training. Longitudinal research could provide insights into the optimal duration and frequency of NMT required to achieve lasting injury prevention benefits.

By addressing these gaps, future research can enhance the understanding of NMT’s long-term effectiveness, guiding coaches, medical staff, and policymakers in developing sustainable injury prevention programs that extend beyond short-term interventions.

## 5. Limitations

This study has several limitations that should be considered when interpreting the findings. First, we cannot ensure that all relevant studies on the topic have been included in this review. Some studies were excluded due to limited access to the full text, and their results might have led to different conclusions. This selection bias may have influenced the overall findings and could limit the completeness of the presented evidence.

Another major limitation is the high variability in results across studies, which is acknowledged but not analyzed in detail. Differences in study populations, intervention protocols, adherence rates, and methodological designs likely contribute to this heterogeneity. However, the absence of a meta-regression or subgroup analysis prevents a deeper understanding of which factors—such as age, competitive level, or gender—most influence the effectiveness of neuromuscular training (NMT). Conducting such analyses in future studies would provide valuable insights into the populations that benefit the most and the conditions under which NMT is most effective.

Regarding the study populations included, most of the analyzed research focused on male athletes, particularly those under 18 years old, even though the inclusion criteria allowed for participants up to 40 years old. As a result, the findings primarily reflect the effects of NMT on adolescent male soccer players, leaving a significant gap in knowledge regarding its effectiveness for female players and older age groups. Given that female athletes have different injury risks—particularly concerning ACL injuries—future research should focus on evaluating NMT effectiveness in women and assessing whether modifications to existing programs could optimize their benefits.

Additionally, the studies included in this review used varying definitions of injuries and different methods for recording them, which may impact the comparability of results. Some studies may have used broader or narrower injury definitions, leading to discrepancies in reported effectiveness. Standardizing injury assessment criteria, such as adopting the UEFA Injury Study system, would improve consistency across studies and allow for more reliable comparisons in future meta-analyses.

Another important limitation is the inconsistency in study designs, including differences in sample sizes, intervention types, duration, and frequency. This heterogeneity makes it difficult to directly compare results and draw definitive conclusions regarding which specific NMT protocol is most effective in preventing lower limb injuries in soccer players. Future research should aim to address these inconsistencies by implementing standardized methodologies and controlled comparisons.

Finally, while the article notes that shorter versions of NMT programs (e.g., 10 min sessions) might be as effective as the standard 20 min versions, this aspect is not explored in detail. A more comprehensive analysis of program duration is necessary to determine the optimal length that balances injury prevention effectiveness with feasibility for coaches and athletes. Future studies should investigate potential trade-offs between shorter and longer protocols, considering factors such as adherence, fatigue, and overall impact on performance.

Addressing these limitations in future research will enhance the understanding of NMT’s impact, improve the standardization of methodologies, and ensure that injury prevention strategies are optimized for different player demographics and training conditions.

## 6. Practical Applications

Neuromuscular training is a program that can significantly reduce the rate of lower limb injuries in soccer players. Thus, it should be widely applied by soccer players. Since many trainers still cling to classical forms of training, strategies should be implemented to inform them about the importance of implementing this type of program and the spectacular results it can have.

Many trainers may have heard about the effectiveness of neuromuscular training, but due to a lack of knowledge they may not be able to design a corresponding program. FIFA 11+ is a ready-made neuromuscular training program that was designed around 15 years ago and is freely available. Moreover, its effectiveness has been tested and proven by many studies. Thus, even if trainers cannot design their own neuromuscular training program, they can implement the one already available. Informing coaches, therefore, should not only focus on informing them about the effectiveness of neuromuscular training, but also where they can find ready-made programs and be informed about the newest developments in the field.

As a shortened version of FIFA 11+ appears to be just as effective as the original 20 min version, it would be important to have this version available online as well, so that coaches can choose the appropriate length of the program according to their needs.

Finally, the study shows that the level of training of coaches in FIFA 11+ is crucial for the effective implementation of the program. Thus, wherever possible, coaches should refer to experts to regularly supervise the training sessions and correct mistakes until both the athletes and the coach are sufficiently familiar, and the maximum possible result is obtained.

## 7. Conclusions

This systematic review confirms that neuromuscular training programs, particularly FIFA 11+, effectively reduce lower limb injury incidence in soccer players. The benefits are enhanced with consistent adherence, proper implementation, and coach education. Female athletes appear to gain more significant injury reduction benefits, particularly in ACL prevention. However, further research is needed to optimize training frequency and duration. Integrating these programs into routine soccer training can enhance player safety and performance, making them an essential component of injury prevention strategies.

## Figures and Tables

**Table 1:** PICO Model: Study Selection Criteria.

Component	Description
Population	Male and female soccer players across different competitive levels
Intervention	Neuromuscular training programs, including FIFA 11+
Comparison	Conventional warm-up programs or no intervention
Outcome	Incidence rate of lower limb injuries

**Table 3:** Quality assessment of the studies based on a quality assessment of selected studies based on the Downs and Black (DB) quality assessment tool scale.

Article	Criteria of Downs and Black (DB) Quality Assessment Tool																											Total Score
	1	2	3	4	5	6	7	8	9	10	11	12	13	14	15	16	17	18	19	20	21	22	23	24	25	26	27	
Soligard et al. (2008) [21]	1	1	0	1	0	1	1	0	0	1	1	1	1	0	1	1	1	1	1	1	1	1	1	1	1	1	2	23
Emery and Meeuwisse (2010) [37]	1	1	1	1	1	1	1	0	0	1	1	1	1	0	1	1	1	1	1	1	1	1	1	1	1	1	1	24
Walden et al. (2012) [36]	1	1	0	1	1	1	1	0	0	1	1	1	1	0	1	1	1	1	1	1	1	1	1	1	1	1	1	23
Grooms et al. (2013) [35]	1	1	0	1	1	1	1	0	1	0	0	0	1	0	0	1	1	1	1	1	1	0	0	0	0	0	0	14
Steffen et al. (2013) [23]	1	1	1	1	1	1	1	0	0	1	1	0	1	0	1	1	1	1	1	1	1	1	1	1	1	1	2	24
Owoeye et al. (2014) [38]	1	1	0	1	1	1	1	0	1	1	0	1	1	0	0	1	1	1	1	1	1	1	1	0	1	1	0	20
Silvers-Granelli et al. (2015) [22]	1	1	1	1	1	1	1	0	1	1	1	0	1	0	0	1	1	1	1	1	1	1	1	0	1	0	1	20
Finch et al. (2015) [39]	1	1	1	1	0	1	0	0	1	1	1	1	1	0	1	1	1	0	0	1	1	1	1	0	1	0	0	18
Silvers-Granelli et al. (2018) [24]	1	1	0	1	0	1	1	0	1	1	1	1	1	0	0	1	1	1	1	1	1	1	1	0	0	1	2	21
Åman et al. (2018) [26]	1	1	1	0	0	1	1	0	0	0	1	0	1	0	0	1	1	1	0	1	1	0	0	0	1	0	0	13
Rahlf and Zech (2020) [25]	1	1	0	1	1	1	1	0	0	1	1	1	1	0	0	1	1	1	1	1	1	1	1	1	1	1	0	21

## Data Availability

Data Availability Statements are available.
